# Development and Implementation of a Bioinnovation Focused Course-Based Research Experience for Undergraduate Students

**DOI:** 10.1007/s43683-022-00099-8

**Published:** 2023-01-19

**Authors:** Leann Norman

**Affiliations:** Department of Biological Sciences, Towson University, Towson, MD 21252 USA

## Challenge Statement

Authentic research opportunities benefit undergraduate students in a variety of ways including increased interest in the STEM field and intent to pursue careers in the sciences. Women and communities of color appear to especially benefit from these types of research experiences.^1,6^ The documentation of authentic research benefits has inspired the development of numerous Course-based Undergraduate Research Experiences (CUREs) at a variety of institutions. CUREs not only assist in the development of short term technical and analytical skills, but also produce long-term outcomes including scientific literacy, aspiration, and identity.^1^ Incorporating authentic research experiences within the classroom may also assist in reducing the stress students typically face when attempting to balance research internships with academic workload.^1,15^ Faculty have also reported a variety of positive themes resulting from the development and adoption of CUREs, including the collection of data that benefits their research program, a way to contribute to promotion and tenure, the development of scientific publications, a method to recruit students into faculty research labs and the creation of stronger student-faculty relationships.^4,10,18^ While CUREs share common goals, research focus of these courses vary greatly, spanning aspects of cell and molecular biology, bioinformatics, and biochemistry among others.^17^ However, there are currently very few CUREs focused on bioengineering and bioinnovation topics. Providing authentic biomedical research experiences in a CURE setting can be challenging due to factors such as semester, class, and instructor time limitations, the broad range of student research experience levels, financial constraints, and developing research projects amenable to a CURE.^18^ An additional barrier to the development of biomedical focused CUREs may arise from the lack of bioengineering resources within a department. The purpose of this paper is to deliver an overview of a one-semester bioinnovation CURE as inspiration and guidance to those at other institutions who are looking to enhance student exposure to authentic bioengineering research.

## Novel Initiative

**Figure 1:**
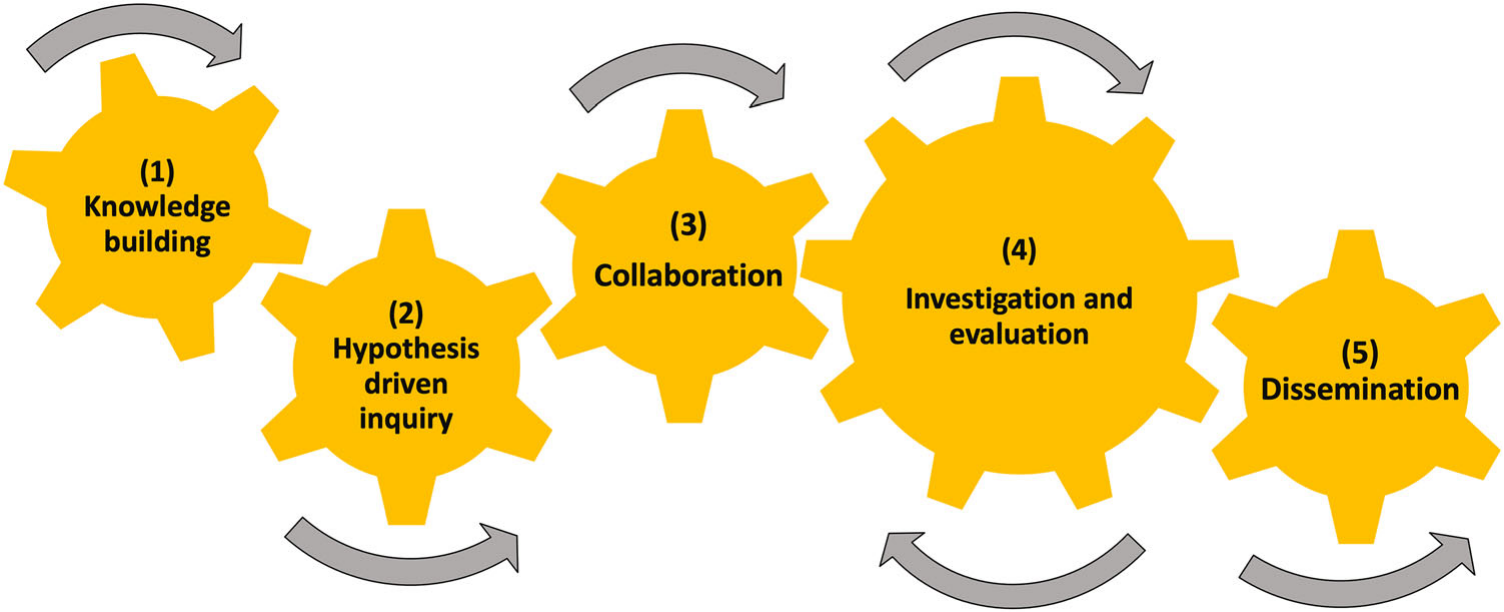
Course design and essential components. Each gear represents a critical component of the class, requiring interactions among each other for the successful completion of the CURE course.

**Figure 2:**
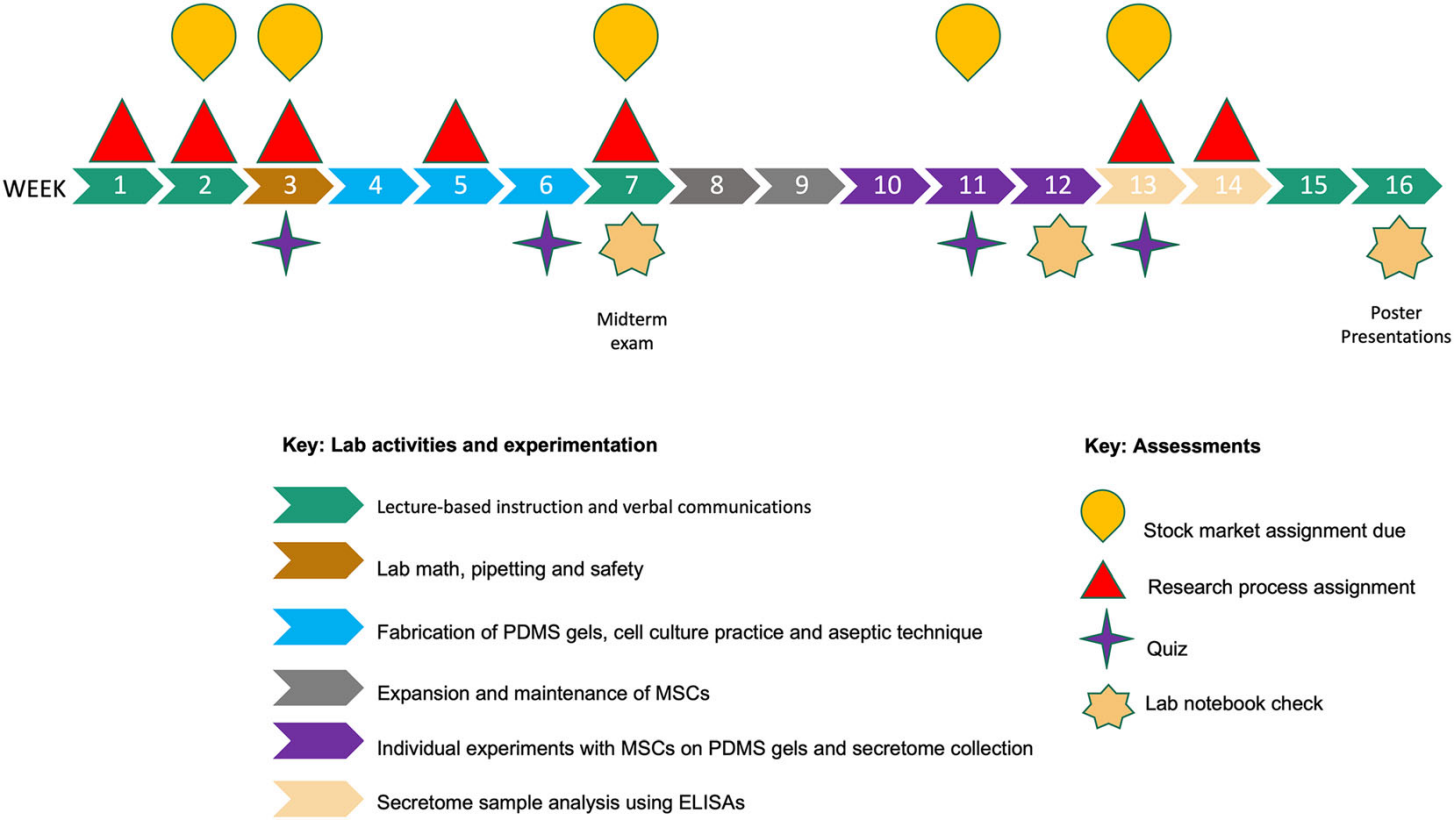
Course timeline and components. The CURE ran during a 16-week period with colored markers illustrating the specific focus of lab activities and experimentation each week. Key assessments are represented by specific symbols throughout the timeline .

CUREs are distinguished from traditional laboratory experiences based on the integration of five dimensions including the use of scientific practices, discovery, broader relevance, collaboration, and iteration.^1^ Rather than modeling a traditional “cookbook” lab, this course was designed to include the five dimensions of a CURE while also integrating the process of bioinnovation over a single semester (Fig. 1). An aim of the course was to encourage students to think about innovative solutions to complex biological problems while performing bioengineering research. Bioinnovation and entrepreneurial topics were embedded throughout the course by combining science and business aspects into the lectures, homework assignments and research design process. Providing an authentic bioengineering research opportunity within the rigid matrix of an academic semester presents a challenge to both the instructor and the students. This paper outlines how a bioinnovation course can be successfully designed to provide an inclusive research opportunity to students within the confines of scheduled class periods. To ensure that students with additional commitments were able to fully participate, research in this course was performed only twice a week during scheduled three-hour class periods. A timeline of course assessments throughout the semester is shown in Fig. 2 with learning objectives and assessment connections shown in Table 1.

**Table 1:** Course learning objectives and corresponding assessment connections.

Learning objective	Assessment connection
(1) Acquire and use basic bioengineering laboratory tools and equipment.	Students were evaluated by observation in the laboratory and lab skill quizzes. (Weeks 2, 3, 5, 7, and 13)
(2) Develop basic aseptic skills needed for mammalian cell culture.	Students were evaluated with lab practical questions on a midterm exam, graded lab notebooks and through laboratory observation. (Weeks 7, 12, and 16)
(3) Design and manufacture scaffolds for use in MSC culture.	Students were evaluated by observation in the laboratory and with graded project design assignments. (Weeks 5, and 7)
(4) Demonstrate proper scientific laboratory record keeping.	Students were evaluated with three periodic notebook collections. (Weeks 7, 12, and 16)
(5) Explain the scientific basis for protein selections and scaffold parameters.	Students were evaluated based on multiple research process assignments. (Weeks 3, 5, 7, and 13)
(6) Quantify MSC secretome proteins using ELISA techniques.	Students were evaluated by observation in the laboratory and through the analysis of experimental results recorded in their lab notebooks. (Weeks 13, 14 and 16)
(7) Search and retrieve information from clinical and financial databases related to the biotech field.	Students were evaluated based on written stock market assignments. (Weeks 2, 3, 7, 11, and 13)
(8) Practice critical thinking skills and apply them to current literature in the field, material presented in lab, and the analysis of lab generated data.	Students were evaluated based on project design assignments and end of semester poster presentations. (Weeks 11, 15, and 16)

### Introduction to the Course and Field

The size of CUREs can range greatly depending on the research, course level, lab space and university size. Many CUREs described in the literature involve smaller class sizes^2,12,16^; however, positive student outcomes achieved in large-enrollment CUREs have also received recent attention.^9,13^ The lab space used for this course had a capacity of 24 students, however, the maximum enrollment was set to 20 students to accommodate space needed for the undergraduate learning assistant (ULA) and instructor. Enrollment in the first iteration of this course included 19 upper-level students majoring in various areas of biology, with successful completion of this course fulfilling an upper-level lab requirement or elective, depending on their concentration. Students were made aware of the new course during open enrollment and upper-level students interested in exploring biomedical research and the biotech industry were encouraged to enroll.

Although students entered the course with a strong biology foundation, most students had minimal experience in cell culture, bioengineering, and regenerative medicine. The first three weeks of the course focused on providing a framework of course expectations while introducing the fields of stem cell biology, biotechnology, and bioengineering laboratory skills. Specific laboratory skills reviewed during this period involved micropipetting, calculating how to prepare solutions and dilutions, aseptic technique, and basic cell culture procedures. Prior to experimentation, students practiced lab skills using dyed water and illustrated aseptic technique using cells from designated practice flasks. This allowed the instructor to provide feedback to the students prior to working with more expensive cells and culture media.

Students were introduced to the biotech industry and bioinnovation through lectures (including entrepreneur guest lecturers and experts in the manufacturing and stem cell fields) and scientific readings that focused on stem cell manufacturing and biomaterials currently used in regenerative medicine. Introducing students to entrepreneurs in the field of regenerative medicine was extremely valuable in creating meaningful connections between the laboratory classroom and the local biotech industry. Since bioinnovation involves not just the scientific research needed to develop a novel biological technology or good, but also the commercialization of these products, the financial world is a critical component to the biotech field. A basic knowledge of finances and investing is invaluable to all entrepreneurs, and to learn more about finances in the biotech sector, students participated in stock market investment simulations through the program Stock-Trak (www.stocktrak.com). StockTrak provides a platform for virtual trading and a class challenge was created for students to individually manage pretend million-dollar portfolios while investing within the biotech and health sectors throughout the semester. Small assignments focused on market basics, investment decisions and the influence of current events on portfolios (Fig. 2).

While learning fundamentals regarding biotechnology and innovation, it was critical for students to become familiar with current applications of stem cells used in regenerative medicine. To narrow the field of regenerative medicine and potential project ideas that students may develop, a focus was placed on learning about mesenchymal stem cells (MSCs) and the MSC secretome. The MSC secretome refers to soluble factors produced by stem cells, including extracellular vesicles such as exosomes, membrane particles and cytokines among other components. Recent emphasis on the MSC secretome and the opportunity to “tune” MSCs for specific therapeutic applications^14^ was the driving force behind this research. During this introductory period of the course, assessments (labeled “Research process assignments” in Fig. 2) were constructed with a focus on various components of the research process, including hypothesis development, specific laboratory techniques and methods, data analysis, and professional networking and communication. A midterm exam was also given to assess student learning (Fig. 2). The first part of the midterm exam required students to micropipette specific volumes of dyed solutions into properly labeled tubes, which were then measured by the instructor. The second portion of the midterm exam required students to calculate standard lab solutions and dilutions, and to respond to questions regarding cell counting, aseptic technique and the development of biomaterials. A successful midterm score illustrated student achievement with learning objectives 1 and 2 (Table 1) and provided a strong foundation for the hypothesis-driven portion of the CURE.

### Project Design

Investigating research questions of interest to the broader scientific community, where the outcome is unknown to both the students and instructor are key characteristics of a CURE course.^1^ In recent years, it has been illustrated that the MSC secretome has very broad-range clinical potential in the field of tissue engineering and regenerative medicine.^14^ Although it’s been well reported that cell behavior is impacted by the extracellular matrix and mechanical cues including stiffness,^3,5^ many questions remain regarding cell-substrate interactions and the effects on secretome composition. By combining these areas of research, new questions surrounding secretome composition and the use of MSCs for tailored disease-specific treatments and additional bioengineering applications can be explored in the laboratory.

In this CURE, students worked in groups to investigate the MSC secretome when cultured under varying conditions. Students were able to modify parameters such as substrate stiffness, cell density, protein coating, cell confinement, pH or other variables if explained appropriately in their project design. Due to financial and equipment limitations, some parameters were implemented, including the requirement to use polydimethysiloxane (PDMS) gels, MSCs and the selection of a cytokine that could be detected through a commercially available enzyme-linked immunosorbent assay (ELISA) kit (DuoSet, R&D Biosystems). Before being allowed to move forward with their experiment, students were required to submit a project design report. The assignment required students to explain their proposed experimental purpose and design in 1-2 paragraphs. Students were specifically asked to incorporate the following questions into their proposal:

1. What part of regenerative medicine (or stem-cell application) relates to your research?
2. How will you use PDMS gels? Will you modify PDMS stiffness or an alternative parameter?
3. Which component of the MSC secretome will you evaluate? Why are you choosing this component and how is it related to regenerative medicine discussed in Question #1?
4. Which variables are you measuring and how do these measurements relate to regenerative medicine discussed in Question #1?

This project design was first done individually to assess each student and their understanding of the connection between an area of regenerative medicine, stem cell application and potential bioinnovation opportunities. Students received instructor feedback after individual assessments and then worked in groups to solidify and confirm a common hypothesis, which needed instructor approval prior to experimentation. Group collaboration was critical in the design process of this course, as it is a prime pedagogical element of a CURE and has been illustrated to improve student communication of biological concepts and ideas.^1^ Students were placed into lab groups of 2–4 people based on the area of regenerative medicine they were most interested in evaluating. Specific areas of interest included cardiovascular disease, bone and tissue injuries, and neurological trauma. Upon group formation, individual projects were designed by the eight groups. Students reviewed feedback received from the individual project design assignment and submitted a new group project design that summarized the same four questions above. While it was not expected for students to have an end “product” within the time frame of the course, students were encouraged to think like entrepreneurs, and to consider how their research would impact the development of a future bioengineered material. The project design assignment stimulated research ideas among students and helped students make connections between their laboratory research and the broader impact. The project design summaries were also used as part of their research poster introductions at the conclusion of the course.

Although details varied among groups, all projects involved the evaluation of MSC secretome when cultured on PDMS gels. The use of PDMS gels allowed students to easily, and affordably, modify substrate stiffness to mimic that of various disease tissue states based on published data.^11^ Students were able to collect the cell secretome at various time points and evaluate the effects of changed parameters on MSC secretion. Example projects involved evaluating the role of IL-6 secretion from MSCs while cultured on PDMS gels of varying stiffness as an optimization parameter for osteonecrosis treatment, the relationship between polydopamine substrate coating concentrations and MSC granulocyte colony-stimulating factor production for enhanced neutropenia therapeutics, and the role of pH level on MSC secretion of IL-6 for bone graft tissue engineering applications.

### Data Collection

Following the midterm exam week, research groups were responsible for their own flasks of MSCs and were required to track confluency and population doubling levels throughout the remainder of the course. To prevent a delay in research timelines due to contamination, a flask of MSCs were also maintained by the instructor. Students continued culturing cells while collecting secretome and measuring samples during the second half of the course (Weeks 8–15 as shown in Fig. 2). Occasionally, separate groups required the collection of secretome samples on different days. To account for these variations, groups were responsible for freezing samples after collection and analysis was performed later as a class. Each student was required to record all experimental methods and analyses in an individual lab notebook. Feedback and grades were given on these notebooks for three separate collections throughout the semester (Fig. 2). The maintenance of student lab notebooks was critical not only in assessing proper scientific laboratory record keeping (Learning objective #4 in Table 1), but also in assisting the instructor in sample identification due to the collection variation among research groups.

Small technical accuracy assignments were created to ensure students could successfully maintain cells in culture, perform accurate cell counts and visualize cells using standard microscopy. A large cell culture lab space, which allowed up to eight students to work simultaneously under the cell culture hood, proved valuable in these assessments. The space ensured that the instructor could both demonstrate and observe successful performance of basic aseptic skills needed for mammalian cell culture (Learning objective #2 in Table 1). Groups were required to submit an assignment that involved developing a data collection calendar which listed the specific dates when they would collect cell secretome and how the tubes would be labeled and stored. These calendars were also transferred to their lab notebooks so that groups could reference the details on their specific collection dates.

Additional research process assignments were also created to encourage students to think about what types of experimental evidence they would need to support or reject their hypothesis. These assignments required students to identify the measurable variables, learn how to source and purchase cytokine detection kits and to explain why this cytokine was selected over other comparable materials. Limiting cytokine detection to specific commercial ELISA detection kits with similar protocols was critical in maintaining class structure and organization. Through lecture and video demonstrations, students were introduced to the ELISA procedures prior to experimentation. Over a two-week period, students performed analysis of their secretome samples using ELISAs (Weeks 13–14 in Fig. 2). A separate individual assignment was created to assess the student’s understanding of the ELISA procedures and how to interpret the data once received. These assignments ended with students preparing figures that were used in final poster presentations.

### Research Ownership

CURES have been shown to increase students’ sense of research ownership when appropriately designed.^7^ In particular, five suggested elements have been described to increase undergraduate student project ownership.^7^ These elements were addressed throughout the course in the following ways:

1. **Students were allowed to make decisions concerning research questions and methods.** *Students selected how to modify their PDMS gels, which component of the secretome to analyze and how to alter the cell culture conditions.*
2. **Students were given the opportunity to pursue an area of regenerative medicine with personal interest.** *Students explored how MSCs are used for therapeutic purposes including cardiac recovery, immunologic diseases, tissue replacement, aging and more.*
3. **The research performed had a broad impact in the scientific community.** *Gathering a more thorough understanding on MSC secretome under varying conditions can provide insight on the optimization of MSCs for therapeutics.*
4. **Students had interaction among peers, professionals, and experts in the field.** *Students worked in groups while researching and troubleshooting experiments. Students were exposed to professionals and experts in the field through guest lectures and end of semester poster presentations.*
5. **Students were involved with projects that demanded higher level problem solving**. *Students were required to justify the selected specific experimental parameters and how they could be used to benefit the field of regenerative medicine.*

At the end of the semester, students were required to create an individual scientific poster. The completion of proper scientific posters illustrated student success with numerous learning objectives, including the practice of critical thinking skills and applications to current literature in the field, material presented in lab and the analysis of lab generated data (Table 1). Students had the option to share at the university sponsored Undergrade Research & Creative Inquiry Poster Session event or individually to the class, since the university sponsored session was held outside of regularly scheduled class time. Most students chose to present at the university poster session. This final “dissemination” gear (Fig. 1) in the course outline provided students with the ability to share with their peers and professors, while also providing an experiential skill for their resume.

## Reflection

### How is this CURE Unique?

The CURE outlined here, while rooted in the background expertise of the instructor, was not designed based on a current research project. The decision to evaluate the MSC secretome was based on the recent attention in the tissue engineering and regenerative medicine fields, as well as the variety of parameters that could be modified during investigation. The intentional design of this broad research area CURE serves as a great framework for future iterations that may involve the exploration of other cell types, biomaterials, or secretome components. It also lends itself to be taught by a variety of instructors with varying levels of bioengineering experience, an advantage when considering the CURE sustainability. The design of this course provided undergraduate students who were not associated with a bioengineering program to perform authentic research in the field while acquiring class credits. By modifying the main research focus to best fit the background knowledge of the student body, it’s possible that this course design could also be adapted for first-year college students, or high-school students, who recently have also been shown to benefit from CURE-based learning.^8^ The course could also be easily adapted for graduate-level students who may be interested in gaining lab experience in the field of bioengineering.

### First Iteration Outcomes

Research focused on CURE assessment mentions that CUREs in their early stages of implementation may be most successful by evaluating how well the program is implemented vs. the evaluation of long-term student outcomes.^1^ Understanding student responses to the first implementation of the course was therefore very valuable. At the end of the semester, students had the opportunity to complete a confidential course evaluation regarding their experiences in the class. Students indicated their level of agreement with a variety of questions related to the course assessments, organization, and learning objectives. Students were asked to select their level of agreement (ranging from strongly agree to strongly disagree) to a variety of prompts including: “I was intellectually challenged by the course” and “Course learning objectives were met”. Of the nine responding students, 100% either “strongly agreed” or “agreed” to these statements. All responding students also strongly agreed to the statements “Assignments/tests reflected the primary content of this course as set out in the course learning objectives” and “Would you recommend this course to others?”.

Students also had the opportunity to leave qualitative feedback regarding the following prompts: “What did you like about this course?”, “What could be improved about this course?” and “Why would you recommend or not recommend this course to others?”. Common responses to the question regarding what students liked about the course included comments that the students gained practical lab skills that can be applied to their future careers, the exposure to primary research and the freedom to experiment. Students further emphasized that they would recommend the course to others because the course gave them valuable experiences and immersed them in the biotech field, while fulfilling a degree requirement. Generally, students were very happy with the challenge that the lab provided, and the experiences learned through the hypothesis development process.

### Course Challenges

By nature, the development of a new course also brought challenges. As previously mentioned, a hallmark characteristic of a CURE is an experimental outcome that is unknown to both the instructor and student.^1^ This uncertainty can often feel risky for instructors as they do not know whether data will be generated or useful.^18^ Although the outcome was unknown, this CURE was designed so that all groups were collecting secretome, detecting a cytokine and therefore acquiring data. Multiple groups were able to see an initial relationship between their measured cytokine and manipulated cell culture parameters. However, due to the 16-week semester timeframe limitation, most groups were only able to report on one set of data and, therefore, were not able to confirm statistical significance at the end of the semester. While there were minimal comments to the response regarding “What could be improved about the course?”, students did mention that more repetition and more time in lab for experiments were desired. It is encouraging that the students enjoyed the lab periods and found them productive, however, increasing the time of the course would potentially create scheduling conflicts with other lab courses and university schedules. A reported faculty benefit from teaching CUREs is the opportunity to recruit students into research laboratories.^18^ In future iterations, it may be possible to encourage those students who are eager to do more research to continue with independent projects after completion of the course. It is also feasible to continue promising projects over multiple CURE iterations, although this would require modifications to the project design portion of the course.

Along the same lines, while one of the goals of the course was to provide an inclusive research setting which encouraged experimentation only during class time, this also presented a challenge since standard cell culture and experimentation is difficult to limit to two three-hour periods per week. To address this challenge, the ULA was critical to the success in the course. The ULA helped with cell maintenance during the days when the class was not scheduled to meet and assisted with occasional sample collection during repeat experiments or data points that needed to be captured outside of schedule lab times. The ULA assistance with cell culture did not appear to impact the students’ ability to effectively learn cell culture and aseptic technique, since students were still able to perform these tasks twice per week. The ULA was also involved in regular lab maintenance, such as waste removal, refilling shared lab solutions, tracking supply inventory and basic lab organization. Prior to their involvement with the course, the ULA had only basic lab experience collected from previous academic coursework. Typically, each week prior to an experiment, the instructor individually trained the ULA on upcoming lab skills so that the ULA was comfortable supporting other students during the scheduled lab periods. For future iterations, it would be ideal to have the ULA selected from a group of former students who successfully completed the course the year prior. Even without prior lab experience, the ULA proved to be a critical component to course success and provided necessary support to both the instructor and class. In addition to lab experience gained during their time serving as a ULA, the individual was also compensated for their hours spent helping in the lab.

Although most experiments were able to be completed during class time, successful completion of the ELISA procedures required the instructor and ULA to perform certain steps outside of the scheduled three-hour window. For the ELISA procedures, students were able to perform approximately 70% of the procedures, while the instructor and ULA completed the remaining portion outside of scheduled class periods. Although the final data was collected by the instructor, the students received their data the following day and worked with the instructor to interpret their ELISA results. Students were also supplied with a supplementary video and protocols illustrating what was done by the instructor and ULA outside of class. Overall, the ULA and instructor served as student support throughout the course, while the students themselves were responsible for conducting the research.

Students were continuously exposed to aspects of bioinnovation and entrepreneurism throughout the CURE and were able to describe how their research would be applied to regenerative medicine and potential therapeutic applications or products during their final poster presentations. However, the end of the semester prevented any progression to a product design or development stage. In future iterations, an aim is to introduce students to university wide entrepreneur programs, venture clubs and innovation challenges which will provide optional innovation opportunities for students who develop a stronger entrepreneurial drive. These specific entrepreneurial introductions may provide students the chance to build on the research they have begun in the classroom and potentially continue that work outside of the laboratory.

Although this course was generously funded by the HHMI Inclusive Excellence Initiative, it is important to mention that the financial cost of developing and teaching a CURE can present a challenge.^18^ The first iteration of the course did require a larger upfront cost, with the cost per student totaling approximately $340. However, many of the purchases, including personnel protective equipment, bulk cell culture supplies, lab equipment and solutions with longer expiration dates will be used again in future classes. The two largest expenses for this course were the individual ELISA cytokine kits and the purchase of human MSCs. These two items accounted for 48% of the total semester expenses. These expenses could have been minimized if students were encouraged to select only from a limited variety of cytokines, if smaller kit volumes were ordered, or if a more affordable cell line was used in place of the human MSCs. Although there are a variety of substrates that would be appropriate to use while evaluating the MSC secretome, PDMS was specifically selected due to its affordability. Expenses were also kept low by using standard cell culture microscopes with the addition of low-cost plug-in eyepiece cameras to capture cell morphology at various stages of culturing, and through the utilization of stock market software already under subscription by the university. As mentioned above, the framework of this course lends itself to adaptation based on the cell types, biomaterials or secretome components of interest, providing flexibility in course expenses as well. Standard lab fees of approximately $90 per student are expected to cover most expenses needed for future iterations of this CURE.

Overall, using the CURE design provided biology students with an authentic research opportunity in the bioengineering field, while simultaneously exploring the fields of bioinnovation and entrepreneurism during scheduled class time. While this CURE focused specifically on stem cells and regenerative medicine, the layout described has the potential to be tailored to the instructor’s background, the resources available in the laboratory and numerous other bioengineering research areas.
